# Substrate‐Driven Differences in Tryptophan Catabolism by Gut Microbiota and Aryl Hydrocarbon Receptor Activation

**DOI:** 10.1002/mnfr.202100092

**Published:** 2021-05-19

**Authors:** Zhan Huang, Tessa Schoones, Jerry M. Wells, Vincenzo Fogliano, Edoardo Capuano

**Affiliations:** ^1^ Food Quality and Design Group Department of Agrotechnology and Food Sciences Wageningen University P.O. Box 17 Wageningen 6700 AA The Netherlands; ^2^ Host‐Microbe Interactomics Group Department of Animal Sciences Wageningen University P.O. Box 17 Wageningen 6700 AA The Netherlands

**Keywords:** aryl hydrocarbon receptor, colonic fermentation, food matrix, short‐chain fatty acids, tryptophan catabolites

## Abstract

**Scope:**

This study aims to investigate the effect of tryptophan sources on tryptophan catabolism by gut microbiota and the aryl hydrocarbon receptor (AhR) activation.

**Methods and Results:**

Four substrates (free tryptophan, soybean protein, single and clustered soybean cells) containing an equimolar amount of tryptophan, but with a different bioaccessibility are studied using in vitro batch fermentation. Tryptophan catabolites are identified by LC‐MS/MS. AhR activity is measured by HepG2‐Lucia AhR reporter cells. The total amount of tryptophan‐derived catabolites increases with decreasing level of substrate complexity. Indole is the major catabolite produced from tryptophan and it is the most abundant in the free tryptophan fermentation. Indole‐3‐acetic acid and indole‐3‐aldehyde are abundantly generated in the soybean protein fermentation. The soybean cell fermentation produced high concentrations of tryptamine. Interestingly, large amounts of short‐chain fatty acids (SCFAs) are also found in the soybean cell and protein fermentation. Both tryptophan‐derived catabolites and SCFAs are able to increase AhR reporter activity over time in all four groups.

**Conclusion:**

This study illustrates that bacterial catabolism of tryptophan and resulting AhR activation in the gut is modulated by the food matrix, suggesting a role for food design to improve gut health.

## Graphical abstract

Microbiota‐directed food is recognized as the trend of healthy food design that targets gut microbiota. This study demonstrates that structural and compositional elements of the food matrix can modulated the fermentative behavior of gut microbiota, resulting in varying concentration, type, and bioactivity of bioactive microbial metabolites.

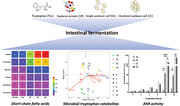

## 1 Introduction

Tryptophan is an essential amino acid and its average daily intake in the western diet is ≈800 mg.^[^ ^1^ ^]^ After digestion and absorption in the small intestine, about 4–6% of the total available tryptophan pass into the colon for microbial fermentation.^[^ ^2^ ^]^ Recently, this fraction of tryptophan catabolized by intestinal micro‐organisms in the colon has gained much interest due to microbial metabolism and formation of aryl hydrocarbon receptor (AhR) agonists, like tryptamine, indoleacrylic acid, skatole, and indole‐3‐aldehyde.^[^ ^3^, ^4^, ^5^ ^]^

AhR is a ligand‐activated regulator of intestinal immunity and it plays a pivotal role in maintaining the balance between intestinal health and disease.^[^ ^6^ ^]^ The specific deletion of AhR in intestinal epithelial cells was shown to significantly induce the development of premalignant lesions.^[^ ^7^, ^8^ ^]^ AhR ligands derived from bacterial catabolism of tryptophan profoundly affect intestinal homeostasis and immune system by promoting gut barrier functions and immune tolerance.^[^ ^9^, ^10^, ^11^ ^]^ Reduced tryptophan metabolism and lower levels of microbial tryptophan catabolites have been measured in patients with various diseases.^[^ ^2^, ^12^, ^13^ ^]^ Available data from both mammalian cell lines and animal models suggest the considerable potential of bacterial tryptophan catabolites activating AhR for prevention and treatment of inflammatory diseases affecting the intestine.^[^ ^12^, ^13^, ^14^, ^15^ ^]^

Tryptophan is most abundant in protein‐rich foods, like meat, fish, and cheese; however, it is also present in plant foods like beans and nuts. Plant proteins have lower digestibility than animal proteins in the upper gastrointestinal tract, due to the presence of antinutritional factors and cell walls.^[^ ^16^, ^17^ ^]^ Therefore a relatively higher fraction of plant proteins than animal proteins, reach the colon. Soybean is one of the most important sources of plant proteins in our diets and it is relatively high in tryptophan content. Recent studies have shown that soybean cell walls can impede access of digestive enzymes to the intracellular proteins resulting in the passage of soybean proteins into the colon.^[^ ^18^ ^]^ Therefore soybean would be a suitable vector for delivering tryptophan to the intestinal micro‐organisms, occurring as particles of varying size, proteins, peptides, or free amino acids.^[^ ^17^, ^19^, ^20^ ^]^

Here we investigated the effect of the food matrix on the production of tryptophan catabolites by human fecal bacteria using a batch fermentation model, as the food matrix is expected to affect the release and availability of substrates to microbiota.^[^ ^21^ ^]^ We used soybean as fermentation substrates and modified the degree of processing of soybean to prepare intact single or clustered soybean cells that were compared with soya protein isolate and free tryptophan, representing different forms in which tryptophan can be delivered to the intestinal micro‐organisms. However, soybean cells also contain large amounts of polysaccharides within the cell wall. Colonic fermentation of these polysaccharides results in the production of short‐chain fatty acids (SCFAs). In addition, SCFAs were reported to have a synergistic effect on the AhR activation,^[^ ^22^, ^23^ ^]^ and butyrate is an AhR agonist in intestinal epithelial cells based on the finding that AhR antagonists and siRNA targeting AhR diminished the expression of AhR regulated genes in presence of butyrate.^[^ ^24^ ^]^ Therefore we also studied the SCFA production during fermentation. The activation of AhR by tryptophan catabolites and SCFAs was performed on HepG2‐Lucia AhR reporter cells.

## 2 Results

### 2.1 Light Microscopy and Particle Size Distribution

**Figure 1:**
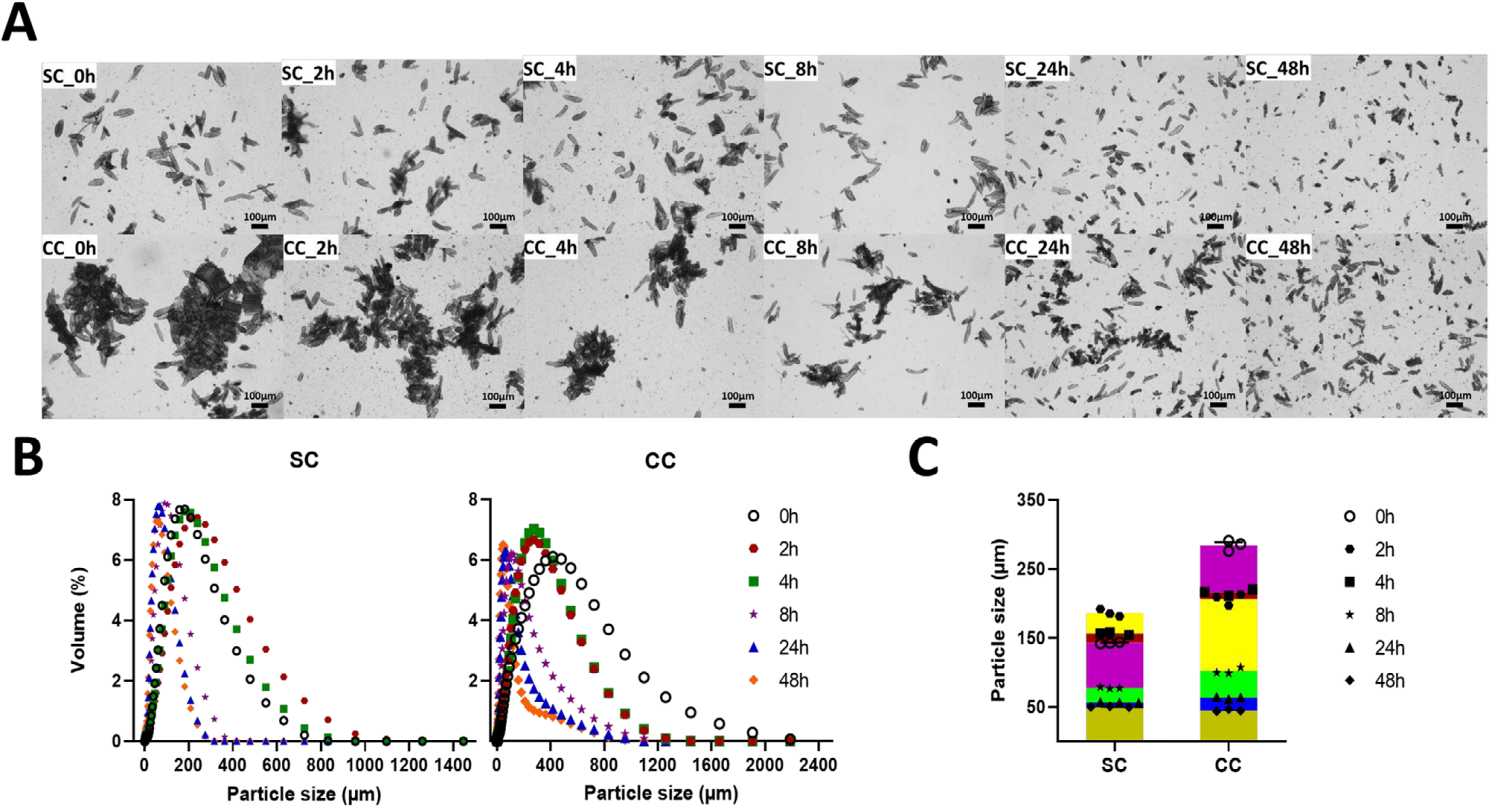
A) Representative light microscopy images, B) size distribution, and C) average particle size of soybean‐based samples collected during fermentation. SC, fermented with single soybean cells; CC, fermented with clustered soybean cells.

The structure of single and clustered soybean cells changed during fermentation (**Figure** **1**). The cell wall limited bacterial degradation in the first 4 h incubation and most cells remained physically intact in the SC group, while clustered soybean cells started to dissociate into smaller particles and single cells (Figure 1A). With longer incubation, the cell particle size decreased reflecting the breakdown of cell structures (Figure 1B,C). Several ruptured cells and possible cell fragments were observed in both groups (Figure 1A), as well as a floating lipid‐like layer due to hydrophobic components that escaped from the cell (data not shown). The breakdown of single cell structure started after 4 h incubation and was almost complete after 24 h, as there were no obvious differences on the average particle size between 0 and 4 h incubation or between 24 and 48 h incubation (Figure 1C). The degradation of clustered cells took longer than single cells and the decrease in the average particle size in the CC group was observed throughout the 48 h incubation (Figure 1C). Even at the end of fermentation, some intact soybean cells were still present (Figure 1A).

### 2.2 Tryptophan and Its Derived Catabolites

**Figure 2:**
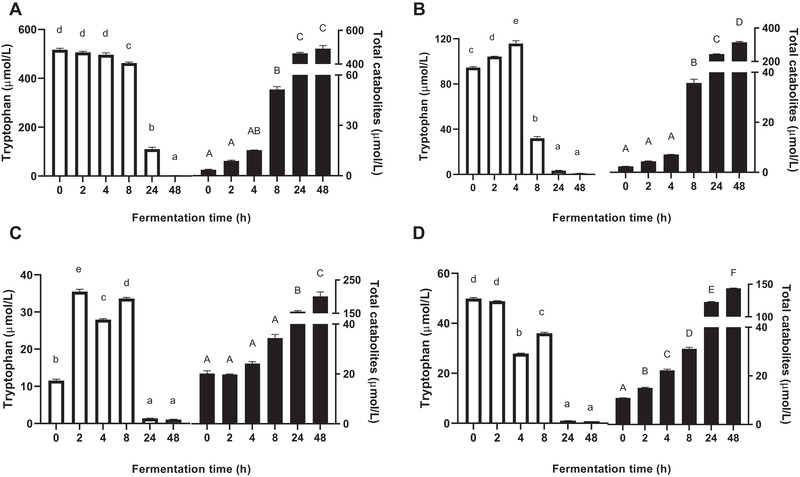
Concentrations of tryptophan (white bars) and total catabolites (black bars) in supernatants collected during fermentation. A) Fermented with tryptophan. B) Fermented with isolated soybean protein. C) Fermented with single soybean cells. D) Fermented with clustered soybean cells. Results are normalized by control samples fermented without supplied substrates and expressed as mean ± SEM (*n* = 3). Different lowercase or uppercase letters above bars indicate significant differences over time (*p* < 0.05, one‐way ANOVA followed by a Tukey post‐hoc test).

**Figure****2** shows changes in the concentration of tryptophan and total catabolites during fermentation. In the Trp group, tryptophan was directly accessible for microbes and its concentration decreased with incubation time (Figure 2A). Conversely, the concentration of tryptophan in the SP group first increased, and then rapidly decreased after 4 h incubation (Figure 2B), due to hydrolysis of proteins and subsequent metabolism by gut bacteria. Similar fluctuations in the tryptophan concentration were observed in the SC and CC groups during the first 8 h incubation (Figure 2C,D). Most tryptophan was catabolized by fecal microbes after 24 h incubation and the catabolites accumulated in the medium. The degradation of tryptophan followed a typical sigmoid kinetics, reflecting a slower rate of formation at the beginning of fermentation and possibly reaching a plateau at longer fermentation times. The concentration of total catabolites, and at the end of fermentation, was the highest in the Trp group, followed by the SP, SC, and CC groups (Figure 2).

**Figure 3:**
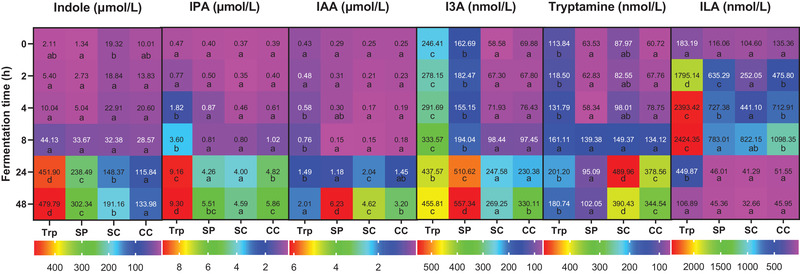
Heatmap of tryptophan‐derived catabolites in supernatants collected during fermentation. Trp, fermented with tryptophan; SP, fermented with isolated soybean protein; SC, fermented with single soybean cells; CC, fermented with clustered soybean cells . Each catabolite has its own legend and corresponding concentrations are normalized by control samples fermented without supplied substrates and presented as the average of three independent replicates. Blocks with different lowercase letters in a row indicate treatments that are significantly different (*p* < 0.05, two‐way ANOVA followed by a Tukey post‐hoc test).

Interestingly the concentration of tryptophan‐derived catabolites in the four groups showed different trends (**Figure** **3**). Indole was the major catabolite produced from tryptophan and its concentration followed the same trend as total catabolites. Large amounts of 3‐indolepropionic acid (IPA) and indole‐3‐acetic acid (IAA) were also found in all groups at the end of fermentation. Relatively small amounts of tryptamine and I3A were produced compared to the other tryptophan catabolite. The Trp group had the highest concentration of indole and IPA after 24 and 48 h incubation. Although less total catabolites were produced in the SP, SC, and CC groups at the end of fermentation, IAA and indole‐3‐aldehyde (I3A) were most abundant in the SP group (*p* < 0.05), and the SC and CC groups contained significantly higher concentrations of tryptamine than the Trp and SP groups (*p* < 0.05). Indole‐3‐lactic acid (ILA) was rapidly generated during the first 8 h incubation, and then further metabolized by microbes, as very limited amounts were found in all groups at the end of fermentation.

### 2.3 Short‐Chain Fatty Acids

**Figure 4:**
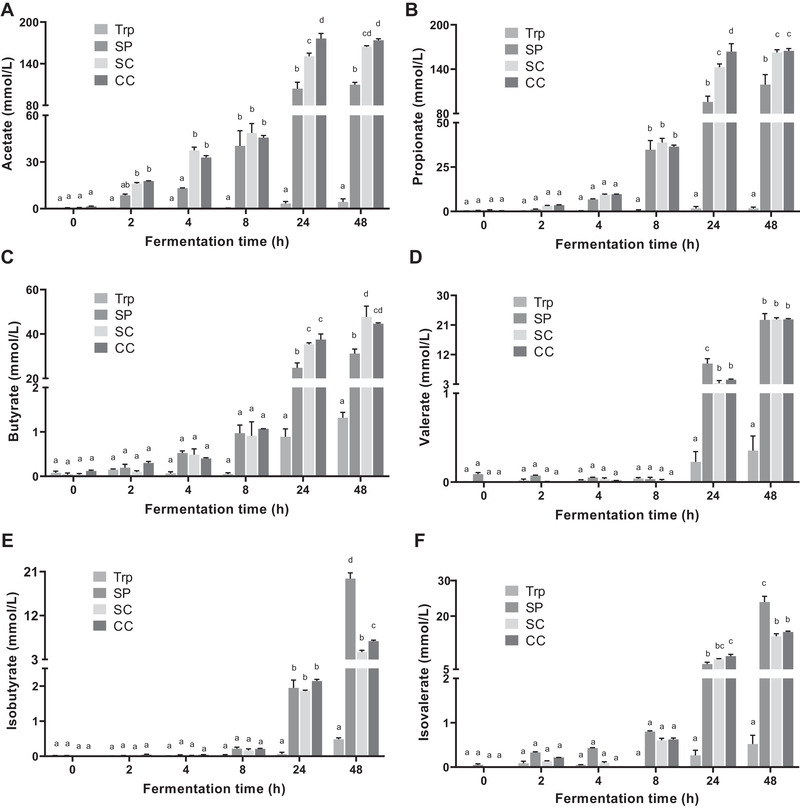
Concentrations of SCFAs in supernatants collected during fermentation. A) acetate; B) propionate; C) butyrate; D) valerate; E) isobutyrate; F) isovalerate. Trp, fermented with tryptophan; SP, fermented with isolated soybean protein; SC, fermented with single soybean cells; CC, fermented with clustered soybean cells. Results are normalized by control samples fermented without supplied substrates and expressed as mean ± SEM (*n* = 3). Bars with different lowercase letters indicate treatments that are significantly different (*p* < 0.05, two‐way ANOVA followed by a Tukey post‐hoc test).

Acetate and propionate were the two major fatty acids produced during fermentation, followed by butyrate, isovalerate, valerate, and isobutyrate (**Figure** **4**). Considerable substrate‐driven changes in the SCFA production were observed in the SP, SC, and CC groups, which all induced high concentrations of SCFAs. Acetate, propionate, and butyrate were significantly produced in the SC and CC groups than that in the SP group after 24 h incubation (*p* < 0.05) (Figure 4A–C). The concentration of valerate in the SP, SC, and CC groups was similar at the end of fermentation (Figure 4D). The SP group yielded the highest concentrations of isobutyrate and isovalerate after 48 h incubation, in comparison to other groups (*p* < 0.05) (Figure 4E,F). Fermentation with single and clustered cells induced similar effects on the SCFA production, but the CC group had significantly higher concentrations of acetate and propionate than the SC group after 24 h incubation (*p* < 0.05) (Figure 4A,B), and significantly high concentration of isobutyrate at the end of fermentation (*p* < 0.05) (Figure 4E).

### 2.4 AhR Activity

**Figure 5:**
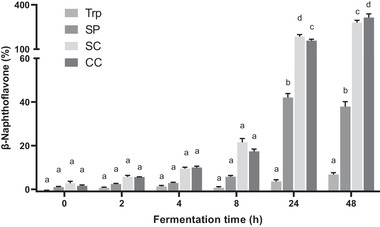
Quantification of the AhR activity of supernatants collected during fermentation using the cell reporter assay. HepG2‐Lucia AhR reporter cells were incubated for 48 h with vehicle (medium or 1% v/v DMSO) and fermented samples. Trp, fermented with tryptophan; SP, fermented with isolated soybean protein; SC, fermented with single soybean cells; CC, fermented with clustered soybean cells. Results are normalized by control samples fermented without supplied substrates and expressed as mean ± SEM (*n* = 3). Bars with different lowercase letters indicate treatments that are significantly different (*p* < 0.05, two‐way ANOVA followed by a Tukey post‐hoc test).

The AhR activity in the four groups increased with incubation time (**Figure** **5**). The Trp group had the lowest AhR activity and this value gradually increased over time. The SC and CC groups exhibited a rapid increase in the AhR activity and showed higher AhR activity compared to the SP and Trp groups. After 24 h incubation, AhR activity in the SP group was significantly higher than that of the Trp group (*p* < 0.05), but markedly lower than that of the SC and CC groups (*p* < 0.05). Interestingly a decrease in AhR activity was observed in the SP group after 48 h incubation. AhR activity in the SC group was higher than that in the CC group except for 48 h, when the CC group gave significantly higher AhR activity compared to the SC group (*p* < 0.05).

**Figure 6:**
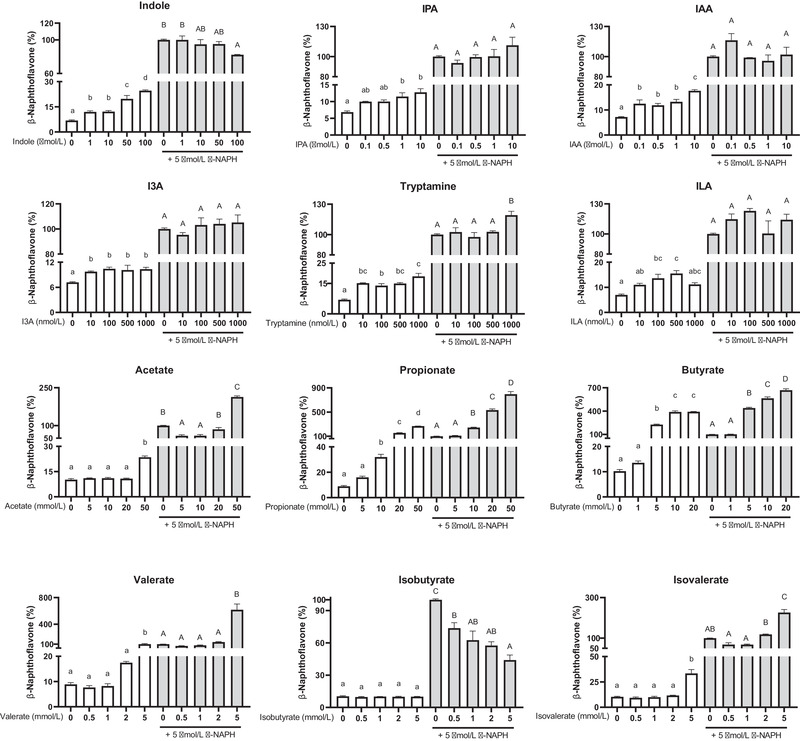
Quantification of the AhR activity of SCFAs and tryptophan‐derived catabolites using the cell reporter assay. HepG2‐Lucia AhR reporter cells were incubated for 48 h with vehicle (medium or 1% v/v DMSO) and increasing concentrations of SCFAs or tryptophan‐derived catabolites, in the absence (white bars) or the presence (gray bars) of β‐NAPH (β‐naphthoflavone; 5 µmol L^−1^). Results are expressed as mean ± SEM (*n* = 3). Bars with different lowercase or uppercase letters indicate treatments that are significantly different (*p* < 0.05, one‐way ANOVA followed by a Tukey post‐hoc test).

To better understand the above results, we further tested the AhR activity induced by tryptophan‐derived catabolites and SCFAs (**Figure** **6**). All detected tryptophan catabolites displayed AhR activity, even at concentrations as low as 10 nmol L^−1^, showing they are much more potent activators of the AhR. In general, AhR activities increased with increasing concentrations of the tryptophan‐derived catabolites. Propionate and butyrate increased AhR activity at concentrations above 10 and 5 mmol L^−1^ respectively. However, we cannot rule out the cytotoxicity of butyrate at high concentrations (above 5 mmol L^−1^) (Figure S2, Supporting Information), as it accumulates in cancer cells growing in glucose.^[^ ^25^ ^]^ The AhR activity induced by acetate, valerate, and isovalerate was found at high concentrations (50, 5, and 5 mmol L^−1^, respectively), whereas isobutyrate had no AhR activity. β‐Naphthoflavone (β‐NAPH) is a potent AhR agonist and was used as the positive control. The β‐NAPH‐induced AhR activation was greatly strengthened in the combination with propionate and butyrate, and slightly increased in the combination with IPA, I3A, tryptamine, and ILA at high concentrations. Acetate, valerate, and isovalerate at low concentrations partially antagonized the AhR activity of β‐NAPH and similar for IAA at high concentrations. Isobutyrate and indole showed the decreasing AhR activity with increasing concentrations, when they were in the combination with β‐NAPH.

## 3 Discussion

To date several studies have been performed to investigate the relationship between tryptophan catabolites and intestinal health,^[^ ^12^, ^14^, ^26^ ^]^ but the dietary factors affecting bacterial catabolism of tryptophan remain largely unknown. This is the first study that investigated the changes in bacterial tryptophan catabolism by ex vivo human gut microbiota using the static batch fermentation model when tryptophan was provided in different forms.

We provided evidence that the incorporation of tryptophan in the increasingly complex structure, that is, in proteins, single cells, or clustered cells modulates tryptophan utilization which can be related to microbial accessibility to tryptophan. As compared to directly available free tryptophan, the intestinal microorganisms needed to secrete enzymes to breakdown soybean cell complexes, degrade the cell wall, and gain access to the intracellular space to break down the protein into amino acids via bacterial proteases and peptidases.^[^ ^27^ ^]^ Therefore, the more complex the structure, the less tryptophan was catabolized, and then the less total catabolites were generated during 48 h fermentation (Figure 2). In addition, the structure of soybean cells also played a role during the early stage of batch fermentation (24 h), in which clustered soybean cells were more rapidly and continuously fermented than single soybean cells. This is possibly due to the junctions between cells providing a microenvironment that was more conducive to the bacterial growth.^[^ ^28^ ^]^ However, this difference in fermentation rate was no longer evident after longer incubation (48 h).

We also found that different substrates alter the type of colonic fermentation and affect the generation of tryptophan‐derived catabolites, likely due to compositional differences among samples. In addition to tryptophan, soybean proteins contain various other amino acids and soybean cell wall contains large amounts of polysaccharides, including pectin, hemicellulose, and cellulose. This means that gut microbiota has a different set of available substrates to grow. In the SP, bacteria actively fermented proteins as energy source which resulted in a pH value above 7 after 48 h fermentation (Figure S1, Supporting Information). In the SC and CC groups, bacteria tend to utilize carbohydrates as energy source rather than proteins, which produces, and is favored by, the significantly lower pH values compared to the control fermentation without supplied substrates (*p* < 0.05) (Figure S1, Supporting Information). The modulation of colonic microbiome induced by shifts toward proteolytic and saccharolytic fermentation altered the bacterial tryptophan catabolism, showing different trends between total catabolites and single catabolites in the four groups.

We further studied the in vitro activation of the AhR during fermentation to gain insight into the outcome of colonic fermentation. In the intestine, several AhR ligands are derived from commensal bacteria, of which the most reported ones are bacterial tryptophan catabolites, such as indole, tryptamine, I3A, IAA, and ILA.^[^ ^2^, ^9^ ^]^ The generation of indole is mediated by tryptophanase that is expressed in many bacterial species, which makes indole the major catabolite derived from tryptophan. ILA can be further converted to IPA by phenyllactate dehydratase and acyl‐CoA dehydrogenase.^[^ ^10^ ^]^ The bacterial tryptophan catabolites activated the AhR, but had varying levels of affinity and reactivity (Figure 6). Tryptamine was found to be a more active AhR activator than IPA, I3A, and ILA. The AhR activity induced by indole was concentration dependent, while indole exhibited an antagonistic effect on the AhR activity in the combination with β‐NAPH. These findings suggest the tryptophan‐derived catabolites contributed to the total AhR activity of fermenter supernatants, but the marked difference in the AhR activity over the time led us to consider other potent ligands affecting AhR activity.

It has been recently shown that SCFAs are able to enhance the activity of other AhR‐active ligands.^[^ ^22^, ^23^, ^29^ ^]^ Consistently, we found large amounts of SCFAs in fermenter supernatants after 24 h incubation in the SC and CC groups, mainly due to the fermentation of polysaccharides within the soybean cell wall by gut microbiota, and in the SP group via the reductive deamination of amino acids (Figure 4).^[^ ^30^ ^]^ In the assay of AhR activity, we confirmed that SCFAs, except isobutyrate, displayed AhR activity especially at high concentrations and they had an synergistic effect on the AhR activation (Figure 6). Isobutyrate had no AhR activity and antagonizes the AhR activation induced by β‐NAPH. The high concentrations of butyrate and propionate inside the fermenter supernatant largely contributed to the significantly high AhR activity of the SC and CC groups after 24 h incubation. As a low concentration of SCFA was found in the Trp group, its AhR activity was mainly induced by tryptophan‐derived catabolites. These results suggest that SCFAs with high concentrations played a more significant role in the total AhR activity of our fermenter supernatants than the tryptophan‐derived catabolites. It is also important to notice that the coverage of AhR activators in this study may have not included all the possible ligands. Some other tryptophan catabolites potentially activating AhR, such as the recently reported indole‐3‐ethanol and indole‐3‐pyruvate,^[^ ^11^ ^]^ were not measured in the present study. It cannot be ruled out that other compounds in the fermenter supernatant might exert agonistic or antagonistic effects on the AhR activation. For a proper interpretation of the AhR activity, it should be considered that the type of cell line used in the assay has an effect on the ligand‐activated AhR activity. Compared with previously reported studies,^[^ ^23^, ^31^, ^32^ ^]^ we observed that the affinity of tryptophan‐derived catabolites and SCFAs for the AhR differ between mice and human cells, as well as in cells from different tissues. This could be due to the sensitivity and transport of tryptophan‐derived catabolites and SCFAs differ in different cell lines. Indole exhibited AhR activity in HepG2 cells, but it was inactive in young adult mouse colonocyte cells.^[^ ^32^ ^]^ Treatment of HepG2 cells with 20 mmol L^−1^ propionate and 5 mmol L^−1^ butyrate gave induction responses similar to β‐NAPH (5 µmol L^−1^), whereas in Caco‐2 cells they induced CYP1A1 mRNA levels less than 5% of TCDD (10 nmol L^−1^).^[^ ^23^ ^]^ In addition, the synergistic effect of acetate, propionate, and butyrate as histone deacetylase inhibitors on AhR responsiveness is also gene‐ and cell context‐dependent.^[^ ^23^ ^]^ Taken together, these results suggest the cell context and reporter gene differences in the AhR activity induced by tryptophan‐derived catabolites and SCFAs.

In summary, our study shows how the form in which a fermented food substrate is provided, and specifically the structural and compositional elements of the food matrix can affect the concentration and type of bioactive microbial metabolites. Importantly, our results may be useful to understand the in vivo fermentation and tryptophan catabolism by gut microbiota, in which the passage time, host metabolism, absorption, and transport of produced metabolites also play a role in the evaluation of overall health effects to the intestine. This study also highlights the need for further studies on the combined effects of different intestinal microbial metabolites on the AhR activation.

## 4 Experimental Section

### Materials and Chemicals

Dried soybean supplied by De Molukken B.V. (batchcode: 3 005 193 871) were purchased from a local supermarket (Wageningen, The Netherlands) and stored at room temperature. Soy protein isolate (91% protein) containing 1.2 g tryptophan per 100 g was purchased from Bulk Powders (Colchester, UK). All chemicals used were purchased from Sigma‐Aldrich (St. Louis MO, USA), unless stated otherwise.

### Sample Preparation

The single and clustered soybean cells were isolated as previously described.^[^ ^18^ ^]^ In brief, soybean was dehulled and autoclaved at 121 °C for 10 min and mashed by Stomacher 400 Circulator (Seward, UK). Soybean cells were isolated using a wet sieve shaker. Particles retained in a sieve with a mesh size between 425 and 250 µm were referred to as clustered cells, and those retained in a sieve between 125 and 71 µm were referred to as single cells. Collected samples were freeze‐dried. The chemical composition of single and clustered cells, as well as soy protein isolate, was determined and presented in Table S1, Supporting Information. The tryptophan content of single and clustered cells was calculated based on their protein content (Table S1, Supporting Information) and assuming a content of 1.311 g tryptophan per 100 g soybean protein.^[^ ^33^ ^]^

### In Vitro Colonic Fermentation

Fresh fecal samples were collected from three Dutch adults, 22–25 years old, with a body mass index (BMI) between 18.5 and 25. All donors were in good health and with no history of gastrointestinal disorders or antibiotic treatment for at least 6 months before this study. Faecal slurries were processed within 2 h after defecation following the method described by Koper et al.^[^ ^34^ ^]^ Batch‐culture fermentation vessels (70 mL working volume) were sterilized and filled with 43 mL autoclaved basal nutrient medium, consisting of 5.22 g L^−1^ K_2_HPO_4_, 16.32 g L^−1^ KH_2_PO_4_, 2.0 g L^−1^ NaHCO_3_, 2.0 g L^−1^ yeast extract, 2.0 g L^−1^ special peptone, 1.0 g L^−1^ mucin, 0.5 g L^−1^_L_‐cysteine HCl, and 2.0 mL L^−1^ Tween 80. Before addition of 7 mL faecal slurries, vessels were flushed with N_2_/CO_2_ (80/20, v/v) gases to create an anaerobic condition. Substrates, containing 10 mg tryptophan, were inoculated with faecal slurries at 37 °C with mild shaking. Fermentation with free tryptophan is referred to here as Trp, with isolated soybean protein as SP, with single soybean cells as SC, and with clustered soybean cells as CC. Fermenter samples were taken at 0, 2, 4, 8, 24, and 48 h after inoculation, and then immediately centrifuged (12 000 g, 5 min) and filtered (0.20 µm regenerated cellulose filter). The supernatant and residue were separately collected and frozen at −20 °C before analysis.

### Light Microscopy

Residues of SC or CC samples from three independent fermentation experiments were defrosted at 4 °C and then combined. The upper layer of sediments, containing mainly products of faecal slurries and some bacteria, were gently scraped. The lower part was diluted with water and covered with a glass coverslip. Microscopy images (5× magnification) were taken with a camera (Axiocam HRc, Carl Zeiss AG, Oberkochen, Germany).

### Particle Size Distribution

The size distribution of SC or CC samples from three independent replicates during fermentation was separately measured by MasterSizer 2000 with laser diffraction (Malvern Instruments, UK). The refractive index of samples was set at 1.473 and that of the dispersant (water) at 1.330. Pump speed was 1200 rpm. Before each test, samples were equilibrated in water for 2 min. All experiments were performed at 25 °C, and each recorded measurement was determined as the average of three scans. The results are expressed as the average of three samples.

### Tryptophan and Its Derived Catabolites

Tryptophan‐derived catabolites in supernatants were quantified via a Shimadzu Nexera XR LC‐20ADxr UPLC system coupled with a Shimadzu LCMS‐8050 mass spectrometer (Kyoto, Japan). Chromatographic separations were accomplished on a Phenomenex Kinetex 1.7 µm EVO C18 100 Å LC column (100 × 2.1 mm) maintained at 45 °C. Mobile phase A was 0.1% v/v formic acid in water and mobile phase B was 0.1% v/v formic acid in methanol. The elution program was as follows: 0–2 min, 0.1% B; 2–6 min 0.1–25% B; 6–10 min, 25–80% B; 10–12 min 80–90% B; 12–15 min 90% B; 15–16 min 90–0.1% B; then re‐equilibration for 8 min. Flow rate was maintained at 0.3 mL min^−1^ throughout the run. 10 µL of sample supernatants were applied to LC/MS/MS analysis. Mass spectrometer was operated using an electrospray ionization source under the positive mode in the multiple reaction monitoring mode with a spray voltage of 4.5 kV. Compounds were identified by comparing with transitions (*m*/*z*) and retention time (RT) of reference standards: tryptophan (*m*/*z* 205.2 → 188.2; RT 2.97 min), tryptamine (*m*/*z* 161.1 → 144.0; RT 2.11 min), indole (*m*/*z* 118.0 → 91.1; RT 9.77 min), indole‐3‐aldehyde (I3A; *m*/*z* 146.1 → 91; RT 9.31 min), indole‐3‐acetic acid (IAA; *m*/*z* 176.1 → 130.0; RT 9.48 min), 3‐indolepropionic acid (IPA; *m*/*z* 190.1 → 130.0; RT 10.35 min), and indole‐3‐lactic acid (ILA; *m*/*z* 206.1 → 118.1; RT 9.01 min). Skatole (*m*/*z* 132.1 → 117.1; RT 11.36 min) and indoleacrylic acid (*m*/*z* 187.9 → 115.1; RT 10.75 min) were also included in the reference, but not found in the fermenter supernatant. Data analysis was performed on LabSolutions LCMS 5.6 (Shimadzu Corporation, Japan). The content of tryptophan catabolites in each group has been normalized by that in the control samples fermented without supplied substrates.

### Short‐Chain Fatty Acids

SCFA measurement was performed using a Shimadzu GC‐2014 (Kyoto, Japan) equipped with a flame‐ionization detector, a capillary fatty acid‐free Stabil wax‐DA column (1 µm × 0.32 mm × 30 m) (Restek, Bellefonte, PA, USA) and a split injector. Supernatants were thawed and combined with the internal standard (0.45 mg mL^−1^ 2‐ethylbutyric acid in 0.3 mol L^−1^ HCl and 0.9 mol L^−1^ oxalic acid) for SCFA quantification. The injection volume was 0.5 µL and the carrier gas was nitrogen. Temperatures of the injector and detector were 100 and 250 °C, respectively. The temperature profile starts at 100 °C, increases to 172 °C by 10.8 °C min^−1^, then to 200 °C by 50 °C min^−1^, and holds for 1 min. Standard solutions of SCFAs (acetate, propionate, butyrate, valerate, isobutyrate, and isovalerate) were prepared and used for identification and quantification. The results were processed using Chromeleon 7.2.10 (Thermo Fisher Scientific, San Jose, CA) and normalized by control samples fermented without supplied substrates.

### AhR Activity

The AhR activity was measured using a luciferase reporter assay method as described previously.^[^ ^35^ ^]^ In brief, HepG2‐Lucia AhR reporter cells (InvivoGen, San Diego, CA) were grown and maintained according to the manufactures instructions. To induce AhR, 20 µL samples separately from three independent replicates and 180 µL cell suspension containing about 20 000 cells were added per well in triplicate to a flat‐bottom 96‐well plate (Corning, USA), which diluted samples by ten times. The plate was placed at 37 °C in a CO_2_ incubator for 48 h, and then 20 µL of stimulated cells supernatant was transferred into a white walled clear bottom 96‐wells plate (Corning, USA), followed by addition of 50 µL QUANTI‐Luc (InvivoGen). The luminescence was immediately measured by a Spectramax M5 (Molecular Devices, USA). The results are expressed as the percentage of AhR activity of positive control (5 µm β‐naphthoflavone or β‐NAPH in DMSO) and normalized by either DMSO, cell growth medium or control samples fermented without substrates.

### Statistical Analysis

Data were expressed as mean ± standard error of the mean (SEM). Statistical analysis was performed by GraphPad Prism 9.1.0 (GraphPad Software, La Jolla, CA). Differences were evaluated using the analysis of variance (ANOVA) indicated in the figure caption. A value of *p* < 0.05 was considered as statistically significant.

## Conflict of interest

The authors declare no conflict of interest.

## Author Contributions

Z.H., T.S., J.W., V.F., and E.C. contributed to the design of this study. Z.H. and T.S. conducted the research. Z.H. wrote the original draft and all authors reviewed and edited the draft.

## Supporting information

Supporting information

## Data Availability

The data that support the findings of this study are available from the corresponding author upon reasonable request.
