# Supplementary material for: Substrate‐Driven Differences in Tryptophan Catabolism by Gut Microbiota and Aryl Hydrocarbon Receptor Activation
Source: Mol Nutr Food Res. 2021 May 19;65(13):2100092. doi: 10.1002/mnfr.202100092 (PMC8365636; doi:10.1002/mnfr.202100092)
Supplement: Supplementary file 1 — Supporting information [file MNFR-65-2100092-s001.doc]

**Supplementary data**

Table S1. Chemical composition^1^ of experimental materials on a dry basis.

| Samples | Protein^2^ (%) | Carbohydrate^3^ (%) | Fat^4^ (%) |
| --- | --- | --- | --- |
| *Soy protein isolate** | 91 | < 1 | 3.3 |
| Single soybean cells | 51.3 | 20.5 | 25.3 |
| Clustered soybean cells | 48.2 | 22.7 | 24.7 |

^1^Results are presented as the average of three replicates, except soy protein isolate* that are from the label

^2^Protein content was determined by DUMAS (FlashSmart N/PROTEIN, Thermo Fisher Scientific)

^3^Carbohydrate content was determined by Total Dietary Fiber Assay Kit (K-TDFR-200A, Megazyme)

^4^Fat content was determined by Soxhlet extraction using hexane as a solvent.

Figure S1. The pH values of supernatants after 48 h fermentation. Control: fermented without substrate; Trp: fermented with tryptophan; SP: fermented with isolated soybean protein; SC: fermented with single soybean cells; CC: fermented with clustered soybean cells . Results are presented as mean ± SEM (n = 3). Bars with different letters indicate treatments that are significantly different (*p* < 0.05, one-way ANOVA followed by a Tukey post-hoc test).





Figure S2. Cytotoxicity of fermented samples, SCFAs, and tryptophan-derived catabolites. HepG2-Lucia™ AhR reporter cells were incubated for 48 h with vehicle (medium) or tested samples. Cytotoxicity was measured by the released lactate dehydrogenase (LDH) using CytoTox 96 Non-Radioactive Cytotoxicity Assay according to the manufacturer’s instructions (Promega, USA). The maximum LDH release (positive control) was achieved by adding10X Lysis Solution. Results are expressed as the percentage of the positive control (100%) with standard error of the mean (n = 3). N.D.: not detected.
